# Incorporating and evaluating citizen engagement in health research: a scoping review protocol

**DOI:** 10.1186/s13643-021-01812-4

**Published:** 2021-09-28

**Authors:** Anmol Shahid, Brianna K. Rosgen, Karla D. Krewulak, Diane L. Lorenzetti, Nadine Foster, Bonnie G. Sept, Jeanna Parsons Leigh, Henry T. Stelfox, Kirsten M. Fiest

**Affiliations:** 1 Department of Critical Care Medicine, Cumming School of Medicine, University of Calgary, Ground Floor, McCaig Tower, 3134 Hospital Drive NW, Calgary, Alberta T2N 5A1 Canada; 2 Department of Community Health Science and O’Brien Institute for Public Health, Cumming School of Medicine, University of Calgary, Calgary, Alberta Canada; 3 Health Sciences Library, University of Calgary, Calgary, Alberta Canada; 4 Dalhousie University, Halifax, Nova Scotia Canada; 5 Alberta Health Services, Calgary, Alberta Canada; 6 Department of Psychiatry and Hotchkiss Brain Institute, Cumming School of Medicine, University of Calgary, Calgary, Alberta Canada

**Keywords:** Citizen engagement, Public participation, Health research, Co-designed research, Models, Frameworks, Health services research, Scoping review, Protocol

## Abstract

**Background:**

Citizen engagement in research is an emerging practice that involves members of the general public in research processes such as priority setting, planning, decision-making, research conduct, implementation, evaluation, and dissemination. Engaging citizens in research, particularly health research, increases the relevance of study findings, minimizes waste by facilitating stewardship over resources, and builds public trust in the research. While several existing frameworks guide the application of citizen engagement principles to health research, it is unclear how citizen engagement can be utilized to maximize benefits and minimize risks and challenges in health research. To address the gaps in knowledge around citizen engagement in health research, we propose a scoping review to synthesize the state of knowledge on methods to incorporate and evaluate citizen engagement in research. A protocol is presented in this manuscript.

**Methods:**

The methodology for our scoping review is guided by Arksey and O’ Malley’s framework for scoping reviews, and additional recommendations by Levac and colleagues. We will include peer-reviewed and gray literature that report on citizen engagement in health research (including biomedical, clinical, health systems and services, and social, cultural, environmental and population health) and report method(s) to conduct, measure, or evaluate citizen engagement. We will systematically search electronic databases (MEDLINE, EMBASE, CINAHL, JSTOR, PsycINFO, Scopus, and Science Direct) from inception onwards and search relevant organizations’ websites for additional studies, frameworks, and reports on citizen engagement. Title and abstract and full-text citations will be screened independently and in duplicate. Data will be extracted independently and in duplicate, including document characteristics, citizen engagement definitions and goals, and outcomes of citizen engagement (e.g., barriers, facilitators).

**Discussion:**

This review will synthesize the definitions, goals, methods, outcomes, and significance of citizen engagement in health research, as well as any potential barriers, facilitators, and challenges outlined in existing literature. The findings will provide an evidence-based foundation for developing new or improved guidance for citizen engagement in health research. Overall, we anticipate that our scoping review will be a preliminary step to meaningful engagement of citizens in research and strengthen the relationship between the scientific community and the public through transparency and collaboration.

**Systematic review registration:**

Open Science Framework https://osf.io/hzcbr.

**Supplementary Information:**

The online version contains supplementary material available at 10.1186/s13643-021-01812-4.

## Background

Citizen engagement in research is defined as the effective and systematic involvement of members of the general public or persons from affected community groups such as patients, caregivers, advocates, and representatives in research processes [1]. Organizations worldwide have suggested that citizen engagement in research may confer benefits to the conduct and uptake of scientific research through improving the relevance of study findings, encouraging the representation of diverse groups in research studies, minimizing waste by facilitating stewardship over resources, promoting mutual learning and understanding, and allowing broad dissemination of research findings beyond traditional academic audiences [2–6]. Most importantly, citizen engagement has demonstrated utility in building public trust in science and research [7]. This has led to citizen engagement becoming a priority for research funding organizations (e.g., the Canadian Institutes of Health Research [CIHR], National Institutes of Health [NIH] in the USA, and the National Institute for Health Research [NIHR] in the UK), policymakers, academic journals, and researchers themselves [1, 8–12]. As a result, citizen engagement is now changing scientific research, particularly health research, to incorporate real-world lived-experiences and perspectives of members of the public.

Engaging citizens in research necessitates researchers to modify their research approach and critically evaluate their work to improve its generalizability and impact. However, while citizen engagement in research has been conceptually substantiated, it is unclear when, for whom, and how it can be most impactful [13]. Despite the availability of frameworks and guidelines for citizen engagement in health research [1, 2], there remains a lack of evidence-based methods to incorporate effective and systematic, or meaningful, engagement and valid measures to assess its impact.

Many studies intending to incorporate citizen engagement struggle to define citizen engagement, adequately describe processes used, discuss findings pertaining to citizen engagement, or share the impact of citizen engagement on their work [14]. To date, reviews and studies on citizen engagement in health research has largely focused on specific diseases, populations, and disciplines [15–20], limiting the generalizability of the findings. This highlights the need for a broad review and synthesis of citizen engagement in health research that could be used to develop a new framework of citizen engagement or enhance and refine existing frameworks with a strong evidentiary basis, ultimately leading to effective and unified citizen engagement strategies.

As the knowledge base on citizen engagement in health research is emerging, we will conduct a scoping review to identify and summarize literature on citizen engagement in health research. Specifically, we will conduct a scoping review to synthesize the state of knowledge on methods to incorporate and evaluate citizen engagement in this field. The outcomes of this scoping review will provide a descriptive summary of the literature on citizen engagement including frameworks, models, and interventions aimed at improving engagement in health research. This will yield foundational results including strengths and weaknesses in existing frameworks, models, and interventions, and help pinpoint areas that can be targeted to improve the relevance and effectiveness of future frameworks and recommendations.

## Methods

This scoping review will be conducted according to recommendations specified by Arksey and O’Malley [21], and enhanced by Levac and colleagues [22]. The review protocol was registered within the Open Science Framework (10.17605/OSF.IO/HZCBR) and is reported in accordance with the Preferred Reporting Items for Systematic Reviews and Meta-Analyses Protocols (PRISMA-P) statement [23] (checklist shown in Additional file 1). The proposed scoping review will be reported in accordance with the Preferred Reporting Items for Systematic Reviews and Meta-Analyses (PRISMA) extension for Scoping Reviews (PRISMA-ScR) [24].

### Stage 1: Identification of the research question

We designed the research question to be broad in order to capture all potentially relevant information sources. This scoping review aims to answer the question: “what is the state of knowledge on methods to incorporate and evaluate citizen engagement in health research?” The proposed research question provides direction to the subsequent steps, and defines the scope of inquiry with respect to population, concept, and outcomes of interest [14].

For the purpose of this scoping review, our target population “citizens” will be defined as consumers of health services, informal caregivers, advocates, and representatives from community organizations, and members of the general public. Our target concept is engagement or participation in health research. For the purposes of this scoping review, we will adopt the CIHR definition of health research, which includes research in the biomedical, clinical, health systems and services, and social, cultural, environmental, and population health fields [18]. We recognize that engagement may take many forms, including but not limited to priority setting, planning, decision-making, research conduct, implementation, evaluation, or dissemination. Additionally, citizen engagement may occur when citizens are joint grant holders or co-applicants on research grants, help to identify research priorities, hold membership in a project advisory or steering group, comment and develop research materials, interact with research participants, and/or help carry out research activities.

### Stage 2: Identification of relevant studies

We will identify relevant literature using a pre-determined plan for data sources and search strategy, including search terms, languages, and dates of search. The search strategies will be designed to maximize the comprehensiveness and breadth of the search, while considering time and personnel workload as limiting factors [14, 15].

#### Search strategy

A search strategy will be developed by the study team, including a medical librarian (DLL), who has led multiple initiatives to develop, adapt, and evaluate approaches to incorporate citizen engagement into research [19–23]. The following databases will be searched from inception: MEDLINE (1879-present), EMBASE (1947-present), Cochrane Library (1996-present), CINAHL (1961-present), PsycINFO (1927-present), Scopus (1970-present), and Web of Science (1964-present). Our search will be broad to encompass relevant terminology in this area, including subject headings and keywords and relevant synonyms related to three concepts: (1) citizens (e.g., community member, lay person, public, stakeholder), (2) engagement (e.g., collaboration, engagement, participation), (3) health research (e.g., biomedical research, health research, public health research). No exclusion criteria will be placed on language, though we will exclude studies published prior to the year 2000. Prior to implementation, the search strategy will be independently reviewed using the Peer Review of Electronic Search Strategies (PRESS) checklist [24]. A draft search strategy (MEDLINE) is shown in Additional file 2.

A targeted search of the gray literature will also be conducted, searching relevant local, provincial, national, and international organizations’ websites and related scientific or national funding organizations (i.e., OpenGrey, Trip, Involve, CIHR, Patient-Centered Outcomes Research Institute [PCORI]) for additional studies, frameworks, and reports on citizen engagement. Finally, the reference lists of included studies and related systematic reviews will also be screened to identify potentially relevant literature in health research.

### Stage 3: Study selection

Following developing a strategy to identify studies, we will screen and select relevant studies for inclusion in the scoping review. As recommended by Levac and colleagues, we will develop inclusion and exclusion criteria a priori, with additional meetings with the study team to refine the study selection process at the beginning, midpoint, and endpoint of the citation screening process in case there are any unforeseen factors to be considered [15].

#### Eligibility criteria

Studies will be eligible for inclusion if they: (1) are primary (e.g., observational or interventional studies) or secondary (e.g., systematic or scoping reviews) research, frameworks, reviews, or reports; (2) report citizen engagement in health research (including biomedical, clinical, health systems and services, and social, cultural, environmental, and population health, as defined by CIHR); and (3) report method(s) to conduct, measure, or evaluate citizen engagement. No restrictions will be placed on language or study design. Studies published prior to the year 2000 will be excluded to capture a current reflection of citizen engagement in health research as citizen engagement is a research concept that has evolved significantly since its inception.

#### Selection process

Retrieved articles will be imported to Covidence (Veritas Health Innovation, Melbourne, Australia) for title and abstract screening, completed independently, and in duplicate by two reviewers. Reviewers will pilot screen the titles and abstracts of 50 articles to ensure consistency of inclusion and exclusion criteria and once a Kappa coefficient of inter-rater agreement ≥ 0.8 is achieved, proceed to screen the remaining articles. If one reviewer indicates an article as potentially relevant at the title and abstract screening phase, the article will proceed to full-text review to ensure inclusivity. Following title and abstract screening, the full text of selected studies will be screened independently and in duplicate by two reviewers. Reviewers will pilot screen the full text of 20 articles, and proceed to screen the remaining articles once a Kappa coefficient of inter-rater agreement ≥ 0.8 is achieved. Both reviewers must agree on inclusion status and reason for exclusion, if relevant. Any disagreements between the reviewers will be resolved by discussion or the involvement of a third reviewer, if required.

### Stage 4: Charting the data

We will chart the data beginning with defining the information to be extracted from the studies by developing an initial data charting form and then refining this form iteratively and through regular discussion with the study team. The study team will develop a standardized data extraction form in Microsoft Excel (version 16.29.1). The data extraction form will be piloted by two reviewers with ten included studies, and revised as needed. Once the final data abstraction form is developed, all relevant articles will then be abstracted independently and in duplicate by two reviewers.

Extracted variables in the data extraction form will include (1) citation details (i.e., author(s), year, country, publisher, document/ research type, (2) participant details (i.e., population of focus, age, sex, gender, geographical location), (3) citizen engagement operationalization (e.g., definitions, type, goals of citizen engagement), (4) study characteristics (i.e., design, sample size, outcome measurement), (5) findings and outcomes of citizen engagement, and (6) any benefits, limitations, challenges, and risks of citizen engagement in the context of health research. A detailed list of variables to be extracted is shown in Table 1.

**Table 1:** Draft list of variables to be extracted

Citation details	List of authors (surnames) Year of publication Country Publisher (e.g., journal, institution) Document type (e.g., framework OR model OR report) Type of research (i.e., primary study OR secondary study) Research area (e.g., health sciences, social sciences, and natural sciences and engineering)	
Characteristics of intended participants (i.e., citizens)	Population of focus (patient, family, healthcare provider, researcher, knowledge-user, decision-maker) Age Sex Gender Geographical location	
Citizen engagement	Operationalization	Definition of citizen engagement Type and goals of engagement (e.g., priority setting, decision-making, research conduct, implementation, evaluation, dissemination) Engagement frameworks used Engagement uptake
	Methods (if applicable)	Study design Sample size Outcome measurement
	Results and discussion (if applicable)	Findings and outcomes of citizen engagement Barriers to citizen engagement Facilitators of citizen engagement Benefits of citizen engagement Challenges and risks of citizen engagement Strategies to mitigate challenges and risks of citizen engagement Author-stated conclusions (if applicable)

### Stage 5: Collating summarizing and reporting the results

Following collation and analysis of the results [21, 22], we will report (i) a PRISMA-ScR checklist to guide reporting and the flow diagram to report the number of unique articles identified, excluded articles, reasons for exclusion, and included articles in the final scoping review [24]; (ii) citation and participant details using descriptive statistics and accompanying interval estimates; (iii) using tables for key characteristics of studies included study findings, methods of citizen engagement presented, and barriers, facilitators, and knowledge gaps. We will accompany the tables with a narrative synthesis of extracted data. The narrative synthesis will also present any limitations of studies included, knowledge gaps identified, and highlight areas that need further research.

### Stage 6: Consultation

We will involve citizens (NF, BS) in study conception and design as well as interpretation and contextualization of the data. This stakeholder consultation (i.e., citizen engagement) will allow for the incorporation of perspectives and insights beyond the literature and is necessary to improve the rigor and uptake of findings [14, 15].

## Discussion

This scoping review aims to synthesize published research, guidelines, and frameworks relevant to citizen engagement in health research. The findings of this scoping review will be applicable to many research disciplines beyond health research such as social sciences research and natural sciences research. The outcome of this scoping review will be a summary of the definitions, goals, methods, outcomes, significance, and evaluation of citizen engagement alongside potential barriers, facilitators, benefits, challenges, or risks.

The potential findings of this scoping review will have several applications. First, we will provide an evidence-based foundation to create novel guidance for citizen engagement in research or enhance existing guidelines, such as those developed by CIHR [1] and NIHR [1, 3]. Second, this scoping review may highlight potential gaps in the literature, directing future areas of study in the science of citizen engagement in health research. Third, our scoping review will act as a comprehensive information source of citizen engagement in research that research teams may learn from and apply to their own work. Fourth, this scoping review will identify where sufficient literature exists to warrant systematic reviews that could inform citizens of their potential to be impactful co-developers of research across many disciplines. Overall, we hope that our study will be a preliminary step to meaningful and effective engagement of citizens in research to improve the impact and uptake of research findings, and strengthen the relationship between the scientific community and the public through transparency and collaboration.

This scoping review will have some limitations. First, due to lack of standardization in terminology and definitions of citizen engagement [25], we may not capture all literature pertaining to the conduct, measurement, or evaluation of citizen engagement. To ensure we capture most relevant literature, we have developed a search strategy with a medical librarian experienced in literature reviews in the health sciences. Additionally, due to intensive NIHR efforts to set standards for patient and public involvement (i.e., citizen engagement) [26], much of the literature on citizen engagement in health research originates from the UK [25, 27–29]. Consequently, our scoping review will likely include more UK-based citizen engagement literature than any other country. To ensure we do not misrepresent worldwide citizen engagement practices, we will clearly state the geographical location of any included studies and highlight any practices distinct to the UK.

### Team preparedness and dissemination of research findings

We are well-positioned to complete this project due to our knowledge and experience in engaging and evaluating the impact of citizen engagement in health research. We have led initiatives to develop, adapt, and evaluate approaches to incorporate citizen engagement into research and routine clinical care through our research programs [19–23]. We have experience with engaging and training patient and family partners in critical care medicine research (e.g., generating ideas, writing grants, priority setting, conducting research, writing manuscripts), and involving patient advisors in communication of research findings to members of the public at open engagement sessions [19–23]. Additionally, we are well-equipped (i.e., large multidisciplinary team comprised of trainees, staff, and senior researchers) to undertake the vast literature this scoping review will capture. We have a track record of high-quality and pertinent systematic and scoping review publications that inform our research [30–33]. Given that data is publicly available (i.e., published articles and online material), we do not anticipate any data-sharing issues in this work.

Any amendments made to this protocol when conducting the study will be outlined in the Open Science Framework protocol and reported in the final manuscript. The findings of this scoping review will be disseminated widely by the study team members through our research team and institutional websites and presentations at local, national, and international conferences. Our citizen research partners will aid in disseminating the study findings (both to lay and scientific audiences), a step we consider critical for this work is to have an impact on citizen engagement in research. We intend to submit the findings for peer-reviewed publication.

## Supplementary Information

**Additional file 1:.** PRISMA-P 2015 Checklist

**Additional file 2:.** Medline Search Strategy

## Data Availability

Not applicable.

## Abbreviations

CIHR: Canadian institutes of health research
NIH: National institutes of health
NIHR: National institute for health research
PRESS: Peer review of electronic search strategies
PRISMA-ScR: Preferred reporting items for systematic reviews and meta-analyses extension for scoping reviews
