# Supplementary material for: Incorporating and evaluating citizen engagement in health research: a scoping review protocol
Source: Syst Rev. 2021 Sep 28;10:260. doi: 10.1186/s13643-021-01812-4 (PMC8480041; doi:10.1186/s13643-021-01812-4)
Supplement: Supplementary file 2 — Additional file 2:. Medline Search Strategy [file 13643_2021_1812_MOESM2_ESM.docx]

Additional File 2. Medline Search Strategy

1. ((citizen* or consumer* or "lay person" or "lay people" or "lay community" or "lay group" or "lay member" or "community member*" or public or stakeholder*) adj3 (involv* or partner* or participation or participatory or collaborat* or contribution* or input or consultat* or engage* or empower* or advisor* or advising)).tw,kf. (26175)
2. Community Participation/
3. Exp Community-Based Participatory Research/
4. Co-design*.tw,kf.
5. 1 or 2 or 3 or 4
6. Exp Biomedical Research/
7. Exp Behavioral Research/
8. Exp Public Health Systems Research/
9. Exp Nursing Research/
10. Exp Health Services Research/
11. ((biomedical or behavio?ral or clinical or medical or population health or public health or nursing or health) adj2 research).tw,kf.
12. 6 or 7 or 8 or 9 or 10 or 11
13. 5 and 12
14. Animals/ not humans
15. 13 not 14
16. Limit 15 to yr="2000 -Current"
